# Thoracoscopic vs open repair of congenital diaphragmatic hernia after extracorporeal membrane oxygenation: a comparison of intra-operative data

**DOI:** 10.1007/s00383-022-05312-x

**Published:** 2023-01-16

**Authors:** Annita Budzanowski, Stavros Loukogeorgakis, Dhanya Mullassery, Simon Blackburn, Joe Curry, Ioannis Ioannou, Usman Ali, Kate Cross, Stefano Giuliani, Paolo De Coppi

**Affiliations:** 1 Great Ormond Street Hospital for Children, SNAPS, London, UK; 2 Zayed Centre for Research, UCL Institute of Child Health London, 30 Guilford Street, London, WC1N 1EH UK; 3 Anaesthesia Department, Great Ormond Street Hospital for Children, London, UK

**Keywords:** CDH, ECMO, Minimal invasive surgery, Neonates

## Abstract

**Purpose:**

ECMO is an escalation treatment for hypoxic respiratory failure in patients with CDH. Open repair has been advocated after ECMO indicating that physiological changes associated to thoracoscopic repair were not well tolerated.

**Methods:**

We have performed a retrospective review of all patients who underwent ECMO prior CDH repair over a 7 year period (2015–2021). Outcome measures were intra-operative Ph, PCO_2_, PO_2_ and FiO_2_ at 30 min, 1 h 30 min, and 2 h 30 min of surgery, operative time and recurrence rate. Data are shown in median (range).

**Results:**

Eleven patients required ECMO prior CDH repair. Six of eleven (55%) were done thoracoscopically (Group A) and five of eleven (45%) via laparotomy (Group B). Two of six (33%) patients (Group A) were converted to a laparotomy, one of six (16%) patient developed a recurrence, and there was no recurrence in Group B. Two of five (40%) patients died within the first 60 days of life, whilst there was no death in Group A. Intra-operative values are shown below.

**Conclusion:**

Whilst this is a preliminary report of a limited number of patients, there is no obvious difference of intra-operative blood gas parameters during surgical repair in patients after ECMO. Thoracoscopic CDH repair may be considered in patients after ECMO.

## Introduction

A Bochdalek hernia is a posterolateral defect in the diaphragm which results due to failure of closure of the pleuro-peritoneal folds and the septum transversum. It can be involved with a varying degree of pulmonary hypoplasia. Extracorporeal membrane oxygenation (ECMO) is an escalation treatment for a reversible respiratory failure and right-sided heart failure in patients with congenital diaphragmatic hernia (CDH) [1].

The aim of the surgical repair is to reduce the content into the abdominal cavity and close the diaphragmatic defect which is done either primarily or with a patch aiming for a tension-free dome-shaped repair. The most common surgical approach is via laparotomy. However, thoracoscopy is increasingly emerging as a valid alternative minimal invasive (MIS) approach. Whilst MIS is still associated to longer surgical time and higher recurrence rate, it is also associated with faster recovery, shorter post-operative intubation, reduced post-operative analgesia and decreased morbidity compared to open procedures [2, 3].

ECMO remains a relative contraindication for thoracoscopic repair. Two main reasons are being discussed in the literature for advocating a laparotomy in patients with CDH after ECMO. First, there is the assumption that patients who required ECMO have a worsen degree of pulmonary hypoplasia and will also have a larger diaphragmatic defect. A thoracoscopic repair will, therefore, be technically more challenging which inevitably will go along with a prolonged procedure time. Further, it has been indicated that physiological changes associated to thoracoscopic repair due to insufflation pressures which result in excessive hypercapnia and acidosis are not tolerated in a neonate post-ECMO [2].

The aim of this study was to compare intra-operative physiological changes in all patients who underwent CDH repair after ECMO comparing thoracoscopic and open procedure.

## Methods

We have performed a retrospective review of all patients who underwent a CDH repair after ECMO over a 7 year period (2015–2021) (Audit number #3216). It is a policy in our institution to perform the surgical repair after the patient has been decannulated from ECMO and reached cardio-respiratory stability. Patients were identified through search of hospital patient record for ‘congenital diaphragmatic hernia’, ‘CDH’ and ‘ECMO’.

Throughout the past years, there has been a gradual shift at our institution in aiming to repair the CDH minimal invasive.

### Thoracoscopic repair technique

The procedure is done with both lungs ventilated. The patient is positioned in a lateral to semi-prone position with the hernia side upwards. We use a 5 mm/30 degree camera and 3 mm instruments. The optical port is positioned just lateral to the tip of the scapula. The working ports are placed in the midaxillary line just below the mamilla and in the paraspinal line. The intra-abdominal content is gradually reduced with blunt instruments. A continuous carbon dioxide insufflation aiming for pressures between 5 and 6 mmHg and a slight head up position serve ideal conditions for this manoeuvre. Once the content is reduced the insufflation pressures are reduced. The diaphragmatic rim is examined aiming not to tatter the muscle fibres. When possible, the diaphragmatic muscle edges are adapted with non-absorbable sutures. If required a patch, preferably Gore Tex, is used, aiming for a dome-shaped tension-free repair of the diaphragm. The posterolateral diaphragm stitches are passed around the ribs and tied extracorporally.

All patients with a posterolateral diaphragmatic defect, who required pre-operative ECMO and subsequently underwent CDH repair were included into the study. Patients who died pre-operatively were excluded. Following data were collected: patient’s gestational age, body weight, side of the defect, age at repair, type of repair, intra-operative Ph, PCO_2_, PO_2_, FiO_2_ and end-tidal CO_2_ (ET-CO_2_) at 30 min, 1 h 30 min and 2 h 30 min of surgery, anaesthetic time, recurrence rate and death.

We used descriptive statistics and performed the analysis of continuous variables with Mann Whitney test. Categorical variables were analysed with Fisher’s exact test. Significance was set at a *P* value of less than 0.05.

## Results

During the study period, we identified 11 patients who required ECMO prior their CDH repair. Six of eleven (55%) were done thoracoscopically (Group A) and five of eleven (45%) via laparotomy (Group B). All patients were prenatally diagnosed.

The babies in both groups were born at similar gestational age (38 weeks) and were repaired at 20 days. Five patients in Group A and three patients in Group B had a diaphragmatic defect on the left side. The babies who underwent an open surgical procedure were smaller (3.3 kg) compared to the thoracoscopic group (3.73 kg; *p* = 0.02).

In keeping with the most severe lung development, all patients required a patch repair. The procedure (anaesthetic time) in the minimal invasive group (Group A) took 270 min and in those patients who underwent a laparotomy (Group B) 210 min. There was no statistical significance (*p* = 0.06). Two of six (33%) patients who underwent a thoracoscopic repair (Group A) had to be converted intra-operatively to a laparotomy due to surgical difficulties related to diaphragm agenesis, which was not related to ventilation. One patient developed a recurrence (Group A). Two of five (40%) patients in Group B died within the first 60 days of life. There has been no death in the thoracoscopic group. (Group A). The median time to follow-up in Group A was 2 months and in Group B was 6 months (Table 1).

**Table 1:** Patient demographics, duration of operation, and post-operative outcomes; data shown in median (range)

	Group A *n* = 6	Group B *n* = 5	*P* value
Sex (M/F)	2/3	4/1	
Gestational age (week)	38 (37–40)	38 (37–39)	0.9
Body weight (kg)	3.73 (3.44–4.27)	3.30 (2.9–3.66)	0.02*
Side of defect (left/right)	5/1	3/2	0.5
Age at time of repair (days)	20 (13–28)	20 (11–26)	0.8
Anaesthetic time (mins)	270 (170–280)	210 (170–250)	0.06
Recurrence	1/6 (16%)	0/5	0.99
Follow-up (months)	2 (0.8–57)	6 (1.3–19)	0.9
Death	0/6	2/5 (40%)	0.2

* *P* < 0.05

Intra-operative pH, PCO_2_, PO_2_, FiO_2_ and ET-CO_2_ at 30 min, 1 h and 30 min and 2 h and 30 min into the general anaesthesia are shown in Table 2. One patient in the minimal invasive group (Group A) had a pH of 6.91 and a hypercapnia with a PCO_2_ of 14.4 kPa recorded at 1 h and 30 min. The patient recovered with a period of stabilisation; the procedure was completed thoracoscopically (Table 2).

**Table 2:** Intra-operative PH, PCO_2_, PO_2_, FiO_2_ and ET-CO_2_ at 30 min, 1 h and 30 min and 2 h and 30 min of general anaesthesia

	Group A *n* = 6		Group B *n* = 5	
Ph		*SD*		*SD*
30 min	7.35	0.22	7.30	0.04
1 h 30 min	7.23	0.18	7.34	0.13
2 h 30 min	7.28	0.15	7.33	0.08
PCO_2_ (kPa)				
30 min	8.01	3.86	8.86	0.85
1 h 30 min	8.37	0.31	8.33	3.12
2 h 30 min	8.29	3.27	8.10	1.98
PO_2_ (kPa)				
30 min	22.01	10.17	29.75	14.63
1 h 30 min	22.48	7.64	25.58	13.82
2 h 30 min	28.90	8.62	19.30	6.25
FiO_2_				
30 min	0.63	0.16	0.60	0.27
1 h 30 min	0.74	0.21	0.59	0.24
2 h 30 min	0.68	0.19	0.52	0.13
ET-CO_2_				
30 min	5.97	2.35	6.50	1.48
1 h 30 min	7.28	1.36	5.80	2.00
2 h 30 min	6.12	0.91	5.86	1.41

**Fig. 1:**
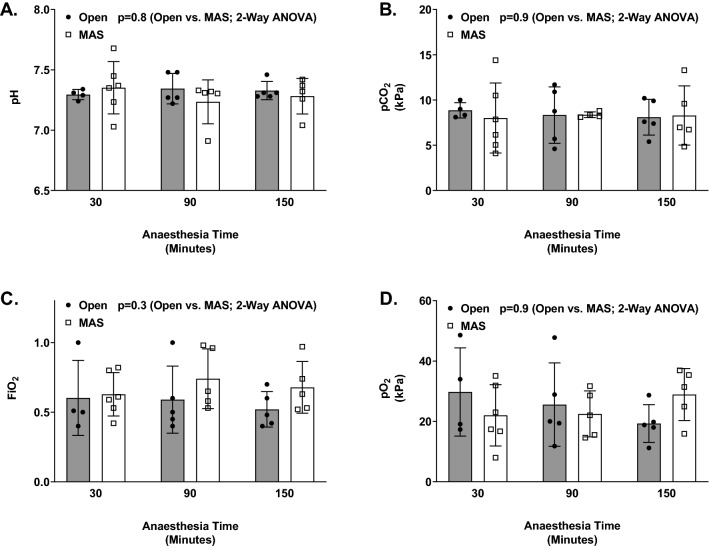
Two-way ANOVA comparison of intra-operative pH, FiO_2_, pCO_2_ and PO_2_ to the anaesthesia time

Two-way analysis of variance (ANOVA) of pH, PCO_2_, PO_2_, FiO_2_ at time intervals 30 min, 1 h and 30 min and 2 h and 30 min into general anaesthesia did not show any significant difference (Fig. 1).

## Discussion

This is a retrospective comparison of intra-operative blood gas parameters in patients with congenital diaphragmatic hernia undergoing thoracoscopic versus open repair.

There is not a consensus on the surgical approach for congenital diaphragmatic hernia repairs. A questionnaire amongst IPEG members demonstrates that 40% are routinely performing a thoracoscopic CDH repair [4]. The majority of open repairs are done via laparotomy. Though there are not any absolute contraindications for thoracoscopic repair, many authors have created their own selection criteria, including position of nasogastric tube, absence of significant cardiac abnormalities, size of the defect, the need for pre-operative ECMO and rib abnormalities [2, 3, 5, 6]. A limited number of successful thoracoscopic repairs have been recently reported in patients post-ECMO [7–9]. Schlager et al. compared a group of patients with CDH post-ECMO done thoracoscopically to a group of patients that were converted to an open procedure. He identified no increase of ET-CO_2_ in those patients who underwent a thoracoscopic repair. Kim et al. reported a series of 15 patients who underwent thoracoscopic repair; 2 patients underwent pre-operative ECMO. One patient had their CDH repaired thoracoscopically and one was converted to an open procedure. Cho et al. compared 29 thoracoscopic CDH repairs to historical open repairs. Two patients in the minimal invasive group had ECMO prior their surgical repair.

Throughout the past years, there has been a gradual shift at our institution in performing them mostly thoracoscopic. Minimal invasive surgery in neonates are technically demanding and advanced procedures with a steep learning curve and are associated with an increased operating time [10]. With technical improvement and increased experience, we have raised our threshold for thoracoscopic repair in patients with CDH. In our study, we have not seen any significant differences between the thoracoscopic group and the open group in regard to side of the defect, anaesthetic time and gestational age. The patient’s body weight was significantly higher with 3.73 kg in the minimal invasive group (Group A) compared to the open group (Group B) with 3.30 kg (*p* = 0.02). We have not set exclusion criteria for MIS in this patient group and despite the shift to minimal invasive surgery within our department the indication for which primary procedure is surgeon specific. A thoracoscopic repair in this group of babies remains a technical and anaesthetic challenge.

Thoracoscopic CDH repair has been associated with shorter ventilation time, reduced requirement of post-operative opiates, better long-term cosmetic outcome and reduced intra-abdominal adhesions [2, 11]. Reasons for conversion from a minimal invasive to an open procedure are large defects, the use of a patch, technical surgical difficulties and increased hypercapnia. Conversion rates vary in the literature from 3.4 to 71%. [5, 7, 8] By some authors, thoracoscopy has been used for assessment of the defect and muscle rim. If a closure without a patch was not feasible, the surgeon would convert to a laparotomy [7]. In our study, all patients in both groups required a patch repair. Two patients (33%) were converted to a laparotomy for technical surgical reasons and not due to ventilation strategies. Both patients had diaphragmatic agenesis with difficulties of a safe reduction of intra-abdominal content. Further, it has been stipulated that thoracoscopic procedures are associated with a prolonged anaesthetic time, hypercapnia and respiratory acidosis due to insufflation of carbon dioxide and the accompanying chest expansion [2]. Bishay et al. demonstrated in a randomised controlled trial comparing thoracoscopic versus open procedures in congenital diaphragmatic hernias and oesophageal atresia, a significant increase in intra-operative hypercapnia and acidosis for patients with CDH without any difference in oxygenation. They found that four out of ten patients experienced a pH < 7 in the thoracoscopic group and no respiratory acidosis in the open group. Within our series, one patient in the thoracoscopic group had a transient hypercapnia (PCO2 > 14 kPa) and respiratory acidosis recorded (pH < 7).

Thoracoscopic repair of diaphragmatic hernia has been associated with increased recurrence rates [7, 12, 13]. There has been a higher recurrence rate in patients with patch repair compared to those who underwent an open repair with a patch as demonstrated from international CDH registry [13]. We had one recurrence in the thoracoscopic group and none in the open group; this did not reach any statistical significance (*p* = 0.99).

We acknowledge the limitations to our study. This is a retrospective review over a 7 year period with a limited number of patients with very complex conditions. Throughout that period, there has been a gradual change not only in surgical technology and innovation but also in the medical management of babies with CDH. With extensive research and continuous evolvement of pre- and post-natal management of these patients, the role and benefit of ECMO remain to be debated [1]. Foetal endoscopic tracheal occlusion (FETO) therapies have shown promise to decrease the need for ECMO in patients with severe cases [14]. A prospective study of the larger cohort is required to endorse our outcome.

## Conclusion

This is a retrospective comparison of intra-operative blood gas parameters in patients with congenital diaphragmatic hernia undergoing thoracoscopic versus open repair. We have not demonstrated any obvious difference of intra-operative blood gas parameters during surgical repair in patients after ECMO. Thoracoscopic repair may be safely considered in patients with congenital diaphragmatic hernia after successful decannulation of ECMO.
